# Safer conception needs of women living with HIV in Southern Ghana: a qualitative study

**DOI:** 10.1177/17449871261419399

**Published:** 2026-03-31

**Authors:** Ellen E Klutsey, Regis Rugira Marie Modeste, Deliwe R Phetlhu

**Affiliations:** Lecturer, Department of Nursing, School of Nursing and Midwifery, University of Health and Allied Sciences, Ho, Volta Region, Ghana; Professor, Department of Nursing and Midwifery, Faculty of Medicine and Health Sciences, Stellenbosch University, South Africa; Professor, Dean of the Faculty of Health Sciences, University of the Free State, Bloemfontein, South Africa

**Keywords:** childbearing, Ghana, safer conception, safer conception needs, safer conception strategies, women living with HIV

## Abstract

**Background:**

Antiretroviral therapy (ART) has improved the quality of life of women living with HIV (WLHIV), increasing their intention and attempts at having children. Educating WLHIV on safer conception strategies helps them to navigate the periconception HIV transmission risk. However, understanding the context-specific needs of WLHIV is crucial in tailoring the provision of SC services. Nonetheless, this is a challenge in Ghana due to information dearth on the subject.

**Aim:**

To explore the safer conception needs of WLHIV who intend to have biological children in the Oti and Volta Regions of Ghana.

**Methods:**

In a qualitative exploratory design, in-depth interviews were conducted with 24 purposively sampled WLHIV who intended to have children within 24 months. Braun and Clarke’s thematic analysis was employed in data analysis.

**Results:**

The needs identified by WLHIV include the need for healthcare worker-initiated communication, education on SC strategies, couple-based education on HIV prevention, continual ARV supply, assistance with fertility, and empathy.

**Conclusion:**

WLHIV have safe conception needs. This finding should inform the organisation of HIV reproductive health and nursing care to ensure a WLHIV-centred service that can facilitate the uptake of SC, thereby contributing to the prevention of periconception HIV transmission.

## Introduction

Women have the right to start families (Amnesty International, 2023). However, for women living with HIV (WLHIV) in sub-Saharan Africa (SSA), having biological children is discriminated against by society and the communities in which they live (Jolle et al., 2022; Matthews et al., 2018). This notwithstanding fertility intentions and childbearing have been documented among WLHIV (Black et al., 2016; Matthews et al., 2018; Tufa et al., 2023; Wekesa and Coast, 2014), raising concerns about the risk of mother-to-child HIV transmission (vertical) and transmission to the sexual partners (horizontal). The prevention of mother-to-child transmission (PMTCT) programmes to fight vertical and periconception HIV transmissions should include services that assist WLHIV with adequate information on safer conception (SC) (Matthews et al., 2018; Mmeje et al., 2014a; Mmeje et al., 2014b).

Safer conception is a combination evidence-based HIV preventive programme with components from the behavioural, biomedical and structural interventions (Davies et al., 2018; Schwartz et al., 2017). It includes the provision of antiretroviral (ARV) therapy for viral suppression, timed unprotected sexual intercourse, timed vaginal self-insemination, provision of pre-exposure prophylaxis (PrEP), treatment of sexually transmitted infections, voluntary male medical circumcision (VMMC), and artificial reproduction termed SC strategies. PrEP involves the preventive use of ARTs by people at risk of HIV infection, whereas VMMC is the surgical removal of the penile foreskin and artificial reproduction limits the direct contact with infected fluids; together, these interventions significantly reduce the risk of HIV transmission. When natural conception is difficult or impossible, artificial reproduction – medical and scientific methods used to assist individuals or couples in conceiving a child – is helpful (Davies et al., 2018; Matthews et al., 2018). A few implementation studies on SC services in Kenya, South Africa, Uganda, and Zimbabwe have reported feasibility, acceptability, effectiveness and uptake among providers and WLHIV (Brown et al., 2016, Brown et al., 2023; Mmeje et al., 2019; Schwartz et al., 2017; Wagner et al., 2021), but it was noted that SC services are not routinely available for in SSA region (Matthews et al., 2018; Mmeje et al., 2016).

Consensus is building in literature that for WLHIV and their partners to be able to attempt pregnancy safely, they need accurate and adequate education on SC strategies (Brown et al., 2016; Crankshaw et al., 2012; Matthews et al., 2018; Mmeje et al., 2012). To do this effectively, it is crucial that what constitutes WLHIV’s needs with regard to the uptake of SC is established to guide service provision that is centred on the WLHIV.

Ghana has a generalised HIV epidemic that is driven mainly through heterosexual sex (Ghana AIDS Commission [GAC], 2016, 2024a). In 2023, Ghana recorded 334,095 people living with HIV (GAC, 2024a), 17,774 new infections, a prevalence range of 0.38–2.12 average at 1.5 (GAC, 2024b). Women are disproportionately affected by HIV infection, with a ratio of 2:1 and reproductive-aged HIV (15–49 years) were estimated at 167,765 accounting for over 50% of the total population of HIV (GAC, 2024b). In 2023, the Oti and Volta Regions had an HIV prevalence of 1.16% and 1.28%, respectively (GAC, 2024c).

Ghana has made modest gains in the HIV response (GAC, 2020). There is a gradual but steady decline in prevalence from 2.2% in 2006 to 1.5 in 2023 (GAC, 2016, 2024a). Being among the 23 priority countries earmarked for the elimination of mother-to-child transmission (UNAIDS, 2011), Ghana’s estimated PMTCT coverage was over 90% in 2023 (GAC, 2023, 2024a). However, Ghana’s reproductive health HIV response is not comprehensive because it does not include SC. The National HIV/AIDS Strategic Plans (from 2016 to 2025) are silent on SC (GAC, 2016, 2020). More so, there is scant literature on SC needs of WLHIV in Ghana (Laar, 2013a, 2013b). This study therefore, explored the SC needs of WLHIV in selected ART units in the Oti and Volta Regions of Ghana.

## Materials and methods

### Study design and setting

The study employed a qualitative exploratory design for this under studied subject. Twelve of the 22 hospitals which were as of 2016, traditionally designated as ART units in the then Volta Region (now split into Volta and Oti Regions in 2019 (Community Water and Sanitation Agency (CWSA), 2023) were used as study sites where participants were recruited. These were the facilities that were operating every weekday and attended to at least six clients a day on average. The Volta Region is in the south-eastern part of Ghana. It is bordered at the south by the Gulf of Guinea and at the north by the Oti Region. Both regions are bounded to the east and west by the Republic of Togo and the Volta Lake, respectively. The Oti Region shares border with the Northern Region (BusinessGhana, 2023; CWSA, 2023).

### Participants

The target population was WLHIV (18–49-year-old) who were receiving HIV care at the traditional ART units in the Oti and Volta Regions. Purposive sampling technique was applied to select WLHIV who participated in the study, and data saturation was achieved and confirmed after 24 interviews when no new information was obtained. Only two of the eligible WLHIV approached declined with the explanation that they were in a hurry. Being on ARVs for at least 6 months and having a desire or attempts at having a child within 24 months were further inclusion criteria. Those WLHIV who were too sick to talk, breastfeeding, or unwilling to give consent were excluded. Breastfeeding WLHIV were excluded because they might not be attempting to conceive at the time of the study.

### Data collection

Using a semi-structured interview guide, face-to-face individual interviews were conducted with the 24 participants between April 2018 to July 2018. The semi-structured interview guide was prepared based on previous studies that investigated facilitators and barriers to SC uptake (Black et al., 2016; Matthews et al., 2014, 2018; Rujumba et al., 2012; Schwartz et al., 2012, 2014, 2016, 2017, 2019; Schwartz and Baral, 2015). The interview guide had two sections, with the first structured section covering socio-demographics and the second section exploring the SC needs of the WLHIV.

Each interview was held in the privacy of the head nurse’s office at the ART units of the selected health facilities. The interview sessions lasted between 25 and 45 minutes and were conducted mostly in Ewe, the predominant dialect of the regions and is also spoken in Togo, Benin, and Nigeria. The interviewer, one of the authors, is a female Ghanaian healthcare professional and a native speaker of Ewe. She is not living with HIV. She shares in the linguistic and cultural background of the participants and that facilitated stronger rapport, more natural communication and a greater sense of trust among participants. It also enabled accurate interpretation of culturally embedded expressions, idioms and non-verbal cues, thereby reducing the likelihood of miscommunication or loss of meaning. However, this insider position might introduce a risk of interpretive bias, as pre-existing cultural assumptions could influence both questioning and analysis; hence, peer debriefing among the authors and co-coding was consistently applied as a control mechanism. Furthermore, the shared identity may also introduce subtle power dynamics or expectations that shape participants’ responses; however, member checking and requests for clarification were done to obtain participants’ actual perspective. Data saturation was reached with 24 participants, as further data collection was not revealing new information.

### Data analysis

All interviews were audio-recorded, transcribed verbatim, and translated into English by an accredited translator. The translated version was matched against the audio-recordings for accuracy, verified by the first author who conducted the interviews to ensure the meaning is maintained, then they were de-identified and participants were given pseudonyms. Data analysis was done manually using inductive thematic analysis as described by Braun and Clarke ( (2006). Open coding was adopted. The data were read and re-read to achieve familiarisation. Descriptive codes were assigned for the first two narratives by all the authors independently. The initial codes were discussed among the authors to reach a consensus which guided the rest of the analysis. The meaningful units of analysis were phrases, sentences, and paragraphs. Similar codes were categorised to form sub-themes and then themes. The themes generated were reviewed by all the authors to ensure that they were representative of the data analysed. Verbatim excerpts from the WLHIV were used to support findings. The conduct of this research followed the consolidated criteria for reporting qualitative research (COREQ) and acceptable practices in the fieldwork, data analysis and interpretation (Tong et al., 2007).

### Trustworthiness of the study

Trustworthiness was ensured through strategies addressing credibility, transferability, dependability, and confirmability. Credibility was strengthened through member check, peer debriefing, and concurrent data collection and analysis, which allowed emerging themes to shape subsequent interviews with WLHIV, as well as through field notes capturing contextual details and non-verbal cues. Furthermore, peer debriefing, discussions, and co-coding by the authors helped in ensuring that the interviewer’s biases are continuously monitored and addressed when needed. Transferability was supported by prolonged engagement and continuing interviews until data saturation, enabling the development of rich, thick descriptions. Dependability was ensured by maintaining a detailed audit trail throughout fieldwork and analysis, documenting all methodological decisions and procedures. Confirmability was enhanced through reflexivity, systematic analytic records, external checks, and the use of field notes to distinguish participant data from researcher interpretation. Additionally, audio-recording, verbatim transcription and certified translation contributed to the rigour and transparency of the data (Ahmed, 2024; Lincoln and Guba, 1985).

### Ethical considerations

Ethical approval was received from the research ethics committee of the University of the Western Cape, South Africa (BM17/6/10) and Ghana Health Service (GHS-ERC:001/11/17). The management of participating institutions also approved the study. The ethics-committee-approved research proposal was strictly followed throughout the study. Data collection was conducted in a private and secure space to ensure participants’ privacy and confidentiality. Pseudonyms were used in all transcripts and reports to maintain anonymity and protect participants’ identities. Interviews were conducted in the participants’ preferred language to remove any risk of discrimination based on language and to promote comfort, accuracy of expression, and respect for their autonomy. The predetermined inclusion criteria were applied consistently, ensuring fairness, justice and the avoidance of any form of discrimination (Miteu, 2024). No compensation was paid to the participants.

### Findings

The participants were within the age range of 23 and 42 years with a mean age of 32 years (SD = 6.2 years). In this study, 88% of the participants indicated that they were citizens of Ghana, and the majority *were of the Ewes tribe* (92%), with at least primary school education (87.8%; Table 1). All the participants were in some form of heterosexual relationship. Only 5 (21%) of the participants knew their partner’s HIV status. On average, the lifetime pregnancies, births and surviving child(ren) were 3, 2 and 1, respectively. Four main themes were identified as per the summary in Table 2.

**Table 1.** Demographic characteristics of the 24 WLHIV.

Characteristic	*n* (%)
Age group (years)	
20–29	10 (41.7)
30–39	10 (41.7)
40+	4 (16.6)
Marital status*	
Married	15 (62.5)
Relationship	9 (37.5)
Nationality	
Ghana	21 (87.5)
Togo	3 (12.5)
Ethnicity	
Ewe	22 (91.7)
Others	2 (8.3)
Level of education	
No formal education	3 (12.5)
Primary	5 (20.8)
Basic education (9–10 years) Secondary school (3 years post-basic)	12 (50.0) 4 (16.7)
Occupation	
Unemployed	4 (16.7)
Employed	20 (83.3)
Duration since HIV diagnosis (years)	
<1	4 (16.7)
1–5	15 (62.5)
6–10	5 (20.8)
Duration of ART use (years)	
<1	6 (25.0)
1–5	14 (58.3)
6–10	4 (16.7)
Lifetime pregnancies	
None	3 (12.5)
1–5	19 (79.2)
6–10	2 (8.3)
Number of pregnancies since tested positive	
None	11 (45.8)
1–5	12 (50)
6–10	1 (4.2)
Lifetime births	
None	6 (25.0)
1–5	17 (70.8)
6–10	1 (25.0)
Children alive	
None	9 (37.5)
1–2	9 (37.5)
3–4	6 (25.0)
Partner status	
Negative	4 (16.7)
Positive	1 (4.2)
Unknown	19 (79.1)

* Marital status (ceremonial and traditionally accepted unions); relationship (cohabiting or courting).

**Table 2.** Themes and sub-themes from the analysis.

Main theme	Sub-themes under the main themes
Confidential and HCW-initiated communication	Ability to approach HCWs on childbearing Time for personal engagement with HCWs Targeted conversation with HCWs One-on-one confidential conversation
Need for education SC strategies	WLHIV’s need for SC education Need for HCWs as teachers of SC strategies
Couple-based education on HIV prevention	Male partner engagement Need for assistance with disclosure
System-driven strategies needed for SC	Need for HCW empathy Need for continual ARV supply for wellness Need for assistance with infertility

ARV: antiretroviral; HCW: healthcare worker; SC: safer conception; WLHIV: women living with HIV.

### Theme 1: Confidential and healthcare worker-initiated communication

The participants need on-going healthcare worker (HCW)-initiated exchange of information on issues of SC aimed at preventing horizontal HIV infection. They look forward to this conversation in an atmosphere of trust and privacy, devoid of judgemental attitude and predispositions. This theme has three sub-themes as discussed below.

#### Ability to approach HCWs on childbearing issues

Some of the participants stated that as they were recently diagnosed with the HIV disease, they felt it was important to approach the HCWs when planning to have a child to make an informed decision that prevents HIV transmission. However, the participants narrated that the reiteration of condom use at every interaction with a HCW sternly prohibits them from initiating any conversation on fertility intentions as indicated in this quote:

Hmmm. That is the problem. So, when I get home, I think about it . . . do they want us to have a child before they say we should use condom? What will happen? I think a lot, but I do not understand. I ask myself (questions). I say one day I will come and ask that if I want to have a child, still should I be using condom or I shouldn’t? (P05, 29 years, partner status unknown)

In the absence of information on SC and inability to approach HCWs on the issue, the participants planned their strategies to have children which were usually contrary to HIV prevention strategies and engagement in HIV care as indicated in the excerpt that follows:

But last time, I said I would like to give birth. So, I said I would not use it so that after it (giving birth) then we can continue (sexual intercourse with condom). . . .giving birth is a priority for me right now. (P10, 41 years, Partner is HIV positive).

A significant number agreed to stopping the use of condoms when they desired to have biological children, without any discussion with the HCWs. Two of the participants admitted to deliberately defaulting follow-up reviews for a refill of the ARVs and resurfaced when they had achieved pregnancy or delivered.

I haven’t reported at the hospital again since I became pregnant until I delivered. So, when the baby was tested and it proved negative, I was asking myself why it was so. I asked myself why I was being wicked towards the baby for it to die (by defaulting antenatal care knowing the baby could be infected). I was wondering why the child did not contract the disease. (P09, 35 years, partner status unknown)

#### Time for personal engagement with HCWs

Another impediment to discussions on SC was the limited time available for HCWs to engage clients on personal reproductive issues during consultations. Participants observed that, although HCWs often faced significant time constraints due to high patient loads, meaningful engagement on matters of procreation remained essential to them. In the absence of such opportunities, some younger WLHIV reported seeking counsel and emotional support on issues related to childbearing from fellow clinic attendees, who were older WLHIV.

So, when I come to the hospital and see some older women with children, I sit beside them. . . The thing that I asked? The child that was born, did it have HIV? . . . I would ask, please can you give me your number? A woman gave me her number, so, I have been making calls with her. Anything (about childbearing), I ask her. (P19, 29 years, partner status is unknown)

#### Targeted and one-on-one confidential conversation

The participants also narrated that their visits to the ART units were full of prescribed service routines. The HCWs focus on running these routine activities and have no time to address any concerns the participants may have as pointed out in this excerpt:

Anytime I come here, lots of people. So, they (attending HCWs) are under pressure there. So, if they ring the bell some people will enter. We do not sit like how we are sitting here now, so I cannot ask this question. Only the questions that are in the book (health record booklet) they would ask me – am I having sex, am I having some pain? Aha. Only those questions I answer; but the question on my mind (on procreation), I did not ask them. (P11, 37 years, partner status unknown)

The participants also indicated that most of the health education sessions were held for all the attendees (all age groups of both sexes) at once. Although the participants admitted that they learnt much during these mass education sessions, many were quick to point out that such a method was not conducive for sensitive discussions like childbearing due to shyness, inability to speak in public, and fear for the HCWs as exemplified in the quotation below:

I could not ask. . . . Some of us feel shy and so cannot speak in public. As you and I are here conversing now, I am talking. But I cannot raise my hand and say anything in public. . . . problems of this nature, I cannot ask. When I come here having issues in my mind, I do not ask. If someone else happens to raise his/her hand and says it, I understand, that is it . . . I cannot ask ooh! (P07, 31 years, partner status unknown)

Furthermore, a few of the participants brought to the fore another dimension of the problem – their inability to initiate a conversation on reproduction with HCWs even if they were accorded privacy, reaffirming a demand for HCW-initiated SC communication.

### Theme 2: Need for education on SC strategies

Most of the participants narrated that they knew they endanger infecting their partners and as such, and worry significantly anytime they had sex without using a condom. This notwithstanding, they reported that they must take this risk to have biological children. This, they said, creates a painful dilemma for them, highlighting their need for education on SC strategies. Almost all the participants admit a state of helplessness and mental stress in this situation.

Yes, there is fear in me (that I might infect my partner) . . . I do not know yet (what to do) . . . They (HCWs) should help me so that if I and the man, (if) we have sexual intercourse and pregnancy ensues, the child should not be infected and the man should also not contract the disease. (P11, 21 years, partner status unknown)

To assuage their fear of infecting their partners, some participants resort to prayer as a coping mechanism. They offered prayers because they believe that whether or not one contracts HIV ‘is an issue of luck’ – an indication of sero-discordance misconception.

I do not know how to handle it (childbearing in HIV). If I should give birth, then we would have to have sexual intercourse without the use of condoms. That is it. We don’t have to use condom; we have to do it without them (condoms). But I have been pondering over it (unprotected sex).. . . My conscience is worrying me about it. So, what I do, (is) I pray that he does not get it. (P13, 32 years, partner status unknown)

Despite concerns about onward transmission to partners while trying to conceive, the participants express their willingness to brave the risk to fulfil their procreation aspirations which they held high. Some of the participants, with subtle openness, asked for advice that can help them conclude as to whether they should bear a child or not.

So, I have to give birth, it will be better . . .. But from my mind, I do not want to do it (to spread the disease). But if you have any advice, you can give it to me; should I do it (have sex which is likely to infect the man) to have a child or I shouldn’t. (P20, 23 years, partner status is negative)

Though a few of the WLHIV outlined how they attempted pregnancy which bears semblance to timed unprotected sexual intercourse (TUI), the precautions needed for its application were always missing in their accounts. None of the participants was able to correctly describe how to determine the ovulation period or indicate that unprotected sex was only allowed at the peak of fertility. Furthermore, all the participants indicated that they were ready to incorporate new teachings on SC to protect their partners and children from being infected with HIV.

I am ready to comply with any advice that will help prevent my husband from getting infected with the disease. I do not know what to do; you have to advise me. (P02, 23 years, partner status is negative)

### Theme 3: Couple-based education on HIV prevention

Fertility decision-making and implementation in heterosexual relationships usually involve both partners; a unity of purpose is even more binding in SC. A couple’s understanding of the dynamics of their serostatuses and how it plays on HIV prevention is crucial to implementing SC strategies successfully. This theme encompasses two sub-themes: male partner engagement and assistance with disclosure.

#### Male-partner engagement

Some participants highlighted a strong need for male-partner engagement in the SC education. They indicated that their male partners usually had the final say in fertility decision-making as per this excerpt:

So, he (partner) keeps saying that even if it is only one child, he would be glad that we have it. For me, I do not want to give birth again; but it is he (partner) who needs a child. That is why we are still pressing on that even if it is one, I bear it for him. (P12, 25 years, partner status is unknown)

To maintain this order, the participants indicated that their partners should be involved in the SC education as they cannot implement the strategies without the approval of their partners. However, the suggested mode of involving their partners differed. Participants who reported disclosure said they intended to visit the facilities with their partners for the HCWs to help orientate them on SC. Others expressed difficulty presenting with their partners and would depend on the ingenuity of the HCWs for couple-based education. A few of the participants indicated that they could relate the SC information successfully to their partners without any difficulty.

The man who is responsible for my fourth pregnancy has no child. When we planned to have that child, we were taught how to use the condom. But I have forgotten what they told us. So, if I cannot educate the guy who wants to marry me, I will ask him to come for a test. When he is tested, he will be counselled on how to use the condom. This will help me to have more education about how the condom should be used before I can get pregnant. (P04, 35 years, partner status unknown)

#### Need for assistance with disclosure

Some participants reported that though they would like to disclose their HIV status to their partners, they need assistance to do so. In a state of non-disclosure, the participants coaxed their partners into using condom under the pretext of preventing pregnancy or spacing their children. Thus, when it is agreed that they need a child, condom use cannot be sustained. Some of the participants recounted that their attempt at disclosure failed and resulted in an accusation of promiscuity. Other anticipated consequences mentioned include divorce, rejection, unplanned disclosure of HIV status, and fear of the unknown. These notwithstanding, some participant felt they need disclose their status to their partners; they desire to visit the health facility with their partners to enable the HCWs assist in the disclosure as expressed in this excerpt:

Hmmm, what I should do, so that the man would be aware of my medication (ARV) and whether he would accept or not. And how I would make him aware (that I have HIV). . . . he may flare up because this disease is not a good one. He could take it to mean I am promiscuous, meanwhile I had my sexual debut very late. . . . I just want you to find a strategy to tell him so that he should know the type of disease I am suffering from which warrants the type of medicine I take. Because whenever he enquires of me as to what disease at all has been found in me that I am on the medication I only answer him that I do not know; and that I was only given the medication. He needs to be told. At my age, I am a grownup; not a kid to be worried if he should say we should break up the relationship. (P22, 42 years, partner status unknown)

### Theme 4: System-driven strategies needed for SC

The participants identified some health system factors that are necessary for them to be able to achieve SC. Three sub-themes were identified.

#### Need for HCW empathy

The participants recounted that when they enquire about some reproductive health issues from HCWs, they were laughed at, bombarded with questions, or scolded as indicated by one of them:

I am not having menstruation. So, the nurse that is attending to me shouted ‘ah! What is it?’ She said that I should go and do the test (pregnancy test). So, I went to do the test, the result was negative. Then another one (nurse) sitting there was laughing at me saying ‘if you are not having menstruation, is it not your pleasure?’ Then the other one (another nurse) said I should not mind her; she is just joking. (P20, 30 years, partner status unknown)

#### Need for continual ARV supply for wellness

Some of the participants believed that they need continual supply and adherence to ARVs to protect their partners against infection. Many of them also recounted that the ARVs would help them in regaining their health to be able to carry a pregnancy successfully. The participants’ reports indicated lack of flexibility and consideration in the provision of ARVs. Instead of encouraging the clients to for optimal adherence, defaulters were refused refills as a punitive measure for skipping their review dates.

So, when I travelled home (hometown) and I was no more using the medication, I was sad. I was not given the medication, as a way of punishment (for defaulting). So today, I came again to plead with them to give me the medication but they said I should come for it later. I know that by the grace of God, I will surely get the medication. (P02, 23 years, partner is negative)

Some of the participants indicated that they believed ARV adherence alone is protective of their partners. Thus, their partners cannot be infected without condom use.

It means that if I do not want him to contract the disease, I have to take the medication (ARVs) because it weakens the virus and prevents the man from contracting the disease. If you do not take the medication, the virus becomes active and by all means, the virus will run and enter the man. So, taking the medication renders the virus too weak to enter the man. (P09, 35 years, partner status unknown)

#### Need for assistance with infertility

Some of the participants indicated that they were experiencing delays with conceiving. They explained that this made them desperate as well as determined to continue trying for pregnancy through protracted unprotected sex. Some also related that in such circumstances, they felt reluctant to negotiate condom use. They also try other means of getting pregnant such as using herbal treatments. This was especially pertinent for those who felt they had advanced in age but did not have children.

The two previous children I gave birth to were unplanned; the pregnancies took me unaware. Then I did not visit the hospital. But now, it is becoming difficult to get pregnant.’ But I am of the view that our inability to have a child stems from my ‘husband’ (she said they were not yet married but cohabiting) himself; he is quite old but has never had a child. (P12, 25 years, partner status is unknown)

## Discussion

In this study, the participants expressed their inability to broach their fertility intentions to HCWs due to the absence of HCW-initiated conversations on the subject. Literature extensively documented that though provider-initiated communication on SC education is an important for the uptake of SC strategies, it is generally lacking (Bekker et al., 2011; Davies et al., 2018; Goggin et al., 2015; Kawale et al., 2015; Matthews et al., 2014, 2015; West et al., 2016). The study findings agreed that there is the need for provider–client conversation that allows for an understanding of the fertility desires/intentions, risk perception, and SC strategies between the two parties. Such conversation also affords the provider the platform to identify opportunities to market SC to those who might not be considering childbearing in the interim (Matthews et al., 2014).

However, studies observed that provider-initiated discussion are not routine services (Finocchario-Kessler et al., 2010; Goggin et al., 2014; Kawale et al., 2015; Matthews et al., 2014; West et al., 2016). HCWs missed opportunities for SC education through reserving such messages with the impression of preventing ‘premature attempted conception’ and deferrals (Matthews et al., 2014; West et al., 2016). Considering that pregnancies are not usually explicitly planned but not unwanted (Davies et al., 2018; Matthews et al., 2013, 2018) those deferred opportunities might be lost as some of the women return to the facility pregnant without the needed SC information (Crankshaw et al., 2012; Wagner et al., 2021).

This study also found that the heavy clinic attendance and the flow of activities were protocol-driven, highly prescribed, and routinised such that there is hardly any room for the participants to express their personal needs. This phenomenon was also described in South Africa (Matthews et al., 2014; West et al., 2016) where it was observed that protocol-driven-care with ready-made messages restrict HIV-care provider’s ability to render tailored messages. From the current study, very few participants approached the providers, mainly due to delayed pregnancy. They had fear of being ridiculed and branded intentional spreaders of HIV. Hence, the WLHIV reported that they consult their fellow clients who are older than them for guidance on childbearing, in an effort to learn from their experiences. Similar observations were noted from other qualitative studies from South Africa, Uganda, and Kenya (Beyeza-Kashesya et al., 2018; Kawale et al., 2014, 2015; Matthews et al., 2014; Mmeje et al., 2016), highlighting the need for HCW-initiated conversation that provides individual attention to the WLHIV. The literature discussed some reasons why WLHIV may not approach providers with childbearing issues; chief among them was anticipated and enacted stigma from HCWs in the form of derogatory remarks and discrimination, regarding their fertility intentions (Kawale et al., 2014; Orza et al., 2015, 2017). This suggests the need for training for providers to be mindful of their attitude towards WLHIV on fertility issues.

Similar to a study conducted in Kenya with those who were in HIV–discordant partnership and had HIV seroconversion (Ngure et al., 2016), in the current study, some participants also expressed the belief that to contract HIV or not was a matter of luck, depicting need for HIV literacy. Despite such misconception, the participants were also willing to learn new HIV prevention strategies that can help them prevent periconception infection. Although, the participants did not know what exactly to expect as assistance from HCWs to aid their effort for SC, they trust that the providers, being professionals, would know as reported in other studies (Matthews et al., 2013, 2015). The participants narrated that they knew they had to protect their partners but endured helplessness because they did not know how to manoeuvre the periconception HIV risk to bear children safely in accord with other studies (Matthews et al., 2013, 2015; Ngure et al., 2016). Confronted by such helplessness and in the absence of SC counselling, some WLHIV sought peer support for childbearing information. Although peer support has been noted as valuable for WLHIV (Goh et al. 2024), it has been noted to be less common with regard to conception and pregnancy desires of WLHIV (Jean et al., 2016). Therefore, the findings of this study emphasise the continued need for SC support and services, similar to other reports from South Africa, Kenya, and Uganda (Gutin et al, 2020; Matthews et al., 2018). Interventions to enhance the implementation of SC services are promising, as post-intervention studies from South Africa and Kenya have reported acceptability, and improved knowledge and attitude towards the SC strategies among persons living with HIV (Brown et al., 2016; Mmeje et al., 2016; Schwartz et al., 2017). It is in this line that the findings of this study provide good grounds for SC education for WLHIV to support them in navigating the periconception HIV transmission risk.

The importance of disclosure in SC uptake and successful implementation is well established (Crankshaw et al., 2012, 2014; Davies et al., 2017). But it was observed in this study that the majority of the WLHIV could not disclose their status due to fear of divorce, being peddled as promiscuous, and isolation. There is therefore the need to help them with disclosure.

This study also highlights the participants need for couple-based education on periconception HIV prevention. Some WLHIV anticipate it could be a platform for the needed disclosure which could relieve them of the secrecy of nondisclosure in accord with other studies (Atuyambe et al., 2014; Maeri et al., 2016; Orza et al., 2015; Rujumba et al., 2012). Some formative SC implementations studies in South Africa and Kenya recorded successes with couple participants (Iyer et al., 2019; Mmeje et al., 2019). Considering that some SC strategies such as timed vaginal self-insemination can best be practised with couple consensus (Crankshaw et al., 2012; Mmeje et al., 2019) disclosure is very important for uptake. This requires that providers are equipped with tact and knowledge via training (Crankshaw et al., 2014; Davies et al., 2017) to enable them to deploy the appropriate disclosure approach that is most suitable for the WLHIV (Bishop and Foreit, 2010; Maeri et al., 2016; World Health Organization, 2012).

For effective provision of SC services, there is a need for system-driven strategies. This study found that WLHIV had the need for continual supply of ARVs for wellness, empathy and assistance with infertility concerns. A number of studies also established that WLHIV need ARV supply and adherence (Davies et al., 2018; Matthews et al., 2012; Schwartz et al., 2014). Engagement in treatment improved the women’s health and they desired and bore children (Wanyenze et al., 2013). SC literature shows that when providers were empathetic towards WLHIV, they communicated a supportive attitude towards their childbearing desires (Kawale et al., 2014; Wanyenze et al., 2013).

Many studies revealed that infertility and its attendant problems are prevalent among WLHIV (Chadwick et al., 2011; Iyer et al., 2019; Nattabi et al., 2009; Schwartz et al., 2017) and need specialist assistance (Davies et al., 2017, 2018). This is because infertility prolongs risky sexual behaviours, heightening the risk of seroconversion when fertility intention exists (Matthews et al., 2018). Considering that childlessness is highly stigmatised as well as being HIV positive, WLHIV face layered stigma which they might go to any length to redress (Finocchario-Kessler et al., 2010; Nattabi et al., 2009; Reidpath and Chan, 2005; Rhodes et al., 2016; Wanyenze et al., 2013).

## Strengths and limitations

This study contributes valuable evidence to an underexplored aspect of HIV care in Ghana by examining the SC needs of WLHIV with fertility intentions. The qualitative design enabled rich, contextual insights, and the use of a culturally and linguistically aligned interviewer enhanced rapport and depth of responses. The detailed description of the study setting and participants, the use of purposive sample of 24 WLHIV and achieving data saturation strengthen the study as they provide opportunity for transferability in other settings.

Nonetheless, the study has limitations. Its focus on the Volta and Oti Regions, coupled with the absence of WLHIV from private ART units – whose managers declined participation – may limit the transferability of the findings to other settings with different cultural or health system contexts. The perspectives captured reflect women in heterosexual relationships, many of whom had not disclosed their HIV status, which may have influenced the narratives provided. Although cultural alignment facilitated communication, it also carries potential for researcher bias. Data were self-reported and cross-sectional, and partner or provider perspectives were not included. Future multi-site, couple-based and quantitative studies which HCWs are needed to broaden and validate the findings.

## Recommendations for practice and future studies

Women living with HIV have interest in SC. SC services in the context of the identified needs will bridge the reproductive healthcare gap for WLHIV with fertility desire and minimise the lost to follow-up due to childbearing intentions. There is the need to study the SC needs of WLHIV in couple-based studies with disclosure for comparison. Research to ascertain punitive measures against ARV defaulters in necessary. Furthermore, quantitative investigations are needed to back up the findings of this study.

## Conclusion

This study highlighted that WLHIV have interest in the uptake of SC, but they have certain needs that should be addressed. In support of their reproductive rights and promoting HIV prevention, SC services should be available and tailored to meet these needs. Awareness creation among nurses and midwives about these needs is important to informing WLHIV-centred nursing care in tackling periconception HIV transmission.

**Key points for policy, practice and/or research**

1. WLHIV are interested in SC to prevent horizontal periconception HIV transmission. Safe conception services should be routinised to enhance accessibility.
2. WLHIV desire HCW-initiated communication on fertility in empathy and privacy placing a demand on capacity building to equip HIV-care providers for this care.
3. WLHIV are denied ARV refill as a deterrent for defaulting, threatening the treatment and prevention principle; there is need for HIV provider reorientation on differentiated service delivery.
4. WLHIV have need for assistance in HIV status disclosure. This service should be made available to facilitate disclosure.
5. Health systems should explore how peer support can be leveraged to strengthen SC services for WLHIV.
